# Association of genetic variations in *GNB1* with response to peginterferon plus ribavirin therapy for chronic hepatitis C in a Chinese population in Taiwan

**DOI:** 10.1186/1471-230X-12-167

**Published:** 2012-11-22

**Authors:** Yun-Ping Lim, Fuu-Jen Tsai, Wen-Ling Liao, Ni Tien, Dong-Zong Hung, Cheng-Yuan Peng, Lei Wan

**Affiliations:** 1 Department of Pharmacy, College of Pharmacy, China Medical University, Taichung, 40402, Taiwan; 2 Department of Emergency, Toxicology Center, China Medical University Hospital, Taichung, 40447, Taiwan; 3 School of Chinese Medicine, China Medical University, Taichung, 40402, Taiwan; 4 Genetic Center, China Medical University Hospital, Taichung, 40447, Taiwan; 5 Department of Pediatrics, China Medical University Hospital, Taichung, 40447, Taiwan; 6 Center for Personalized Medicine, China Medical University Hospital, Taichung, 40447, Taiwan; 7 Department of Laboratory Medicine, China Medical University Hospital, Taichung, 40447, Taiwan; 8 Department of Veterinary Medicine, National Chung Hsing University, Taichung, 40227, Taiwan; 9 Department of Medical Laboratory Science and Biotechnology, China Medical University, Taichung, 40402, Taiwan; 10 Department of Internal Medicine, China Medical University, Taichung, 40402, Taiwan; 11 Department of Internal Medicine, Division of Digestive System and Gastroenterology, China Medical University Hospital, Taichung, 40447, Taiwan; 12 Department of Health and Nutrition Biotechnology, Asia University, Taichung, 41354, Taiwan

**Keywords:** Hepatitis C virus, Standard of care treatment, Rapid virological response, G protein

## Abstract

**Background:**

The aim of this study was to evaluate whether polymorphisms in the guanine nucleotide binding (G protein), beta polypeptide 1 (*GNB1*) gene are associated with a rapid virological response (RVR) among HCV genotype 1 (HCV-1) and 2 (HCV-2) infected patients receiving peginterferon plus ribavirin treatment (PEG-IFNα-RBV).

**Methods:**

We analyzed the association between RVR to PEG-IFNα-RBV therapy and 4 tagging single nucleotide polymorphisms (SNPs) of the *GNB1* gene. This study included 265 HCV-1 and 195 HCV-2 infected patients in a Chinese population in Taiwan.

**Results:**

Among the *GNB1* SNPs examined, the combination of genotypes G/G and G/T populations of rs12126768 was significant inversely correlated with RVR in HCV-1 infected patients (*P* = 0.0330), whereas HCV-2 infected patients, combination of A/A and A/C genotypes populations at rs4648727 responded better to the PEG-IFNα-RBV treatment (*P* = 0.0089). However, there were no significant differences in the allele frequencies of those SNPs between RVR responders and non-responders. Several RVR susceptibility *GNB1* haplotypes were identified, and the ACAT haplotype of the 4 SNPs may increase the successful outcomes of HCV-1 and HCV-2 infected patients (*P* = 0.0261 and *P* = 0.0253, respectively).

**Conclusion:**

The data for *GNB1* SNPs and the association of RVR showed that *GNB1* polymorphisms might be associated with the therapeutic outcomes of HCV-1 and HCV-2 infected patients under standard of care (SOC) treatment.

## Background

Chronic hepatitis C virus (HCV) infection remains a major public health concern, with approximately 200 million individuals, that is, 3% of the world population, infected [1]. Only 20–30% of HCV infected people will recover spontaneously, while the majority of infected populations with persistent infection may subsequently develop liver fibrosis, liver cirrhosis (LC), and hepatocellular carcinomas (HCC) [2]. Therefore, successful treatment is extremely important.

The standard of care (SOC) for HCV infection consisted of pegylated interferon (PEG-IFN)-α 2a or 2b plus the nucleotide analog, ribavirin (RBV) [3]. However, there are variations in treatment outcome among different populations. The genotype and viral load of HCV infected patients are 2 major factors that determine treatment outcome. Patients infected with HCV genotype 2 and 3 (HCV-2/3) achieved an 80% HCV eradication rate; however, only about 50% of individuals infected with HCV genotype 1 (HCV-1) achieve eradication [3]. Successful treatment, meaning no detectable HCV RNA after 6 months of complete therapy, is termed as sustained virological response (SVR). At the beginning of treatment, a rapid virological response (RVR, HCV RNA negative at week 4) is the strongest predictor of SVR [4].

Under SOC treatment, several significant differences in therapeutic response due to ethnic effects have been reported. HCV-1 infected Caucasian patients achieve a better SVR rate than African American patients (40–52% vs. 20–28%), while HCV-2/3 infected Caucasian patients also achieve a better SVR rate than African Americans patients (82% vs. 57%) [5,6]. Therefore, host genetic factors may play a role in the natural route of infection as well as treatment outcome.

Several host genetic factors that may be associated with the efficacy of IFN therapies have been reported, including mannose-binding lectin (MBL) [7], myxovirus resistance protein A (MxA) promoter [8], low-molecular-mass polypeptide 7 (LMP7) [9], apolipoprotein E4 [10], interleukin-10 (IL-10) promoter [11], IFNα receptor 1 (IFNAR1) [12], cytotoxic T-lymphocyte antigen 4 (CTLA4) [13], RANTES [14], interleukin-12 (IL-12) [15], osteopontin (OPN) [16], G protein β3 subunit (GNB3) [17], mitogen-activated protein kinase-activated protein kinase 3 (MAPKAPK3) [18], and interleukin 28B (IL-28B) [19]. Although some of above genes are indirectly correlated with the IFN pathway, the involvement of polymorphisms in genes encoding components of the chemokine system may significantly influence treatment response, for example, the *GNB3* gene.

G protein acts as a key regulator of several membrane-mediated signal transduction cascades [20]. Different kinds of signaling that trigger G protein activation lead to dissociation of the trimeric form of G protein into the Gα subunit and the Gβγ complex. Typically, the Gβγ subunits cannot dissociate, except through non-denaturing conditions [20]. The Gβγ subunits act as a functional unit that controls numerous essential cell functions such as gene activation and repression [21]. Although the interferon signaling cascades do not directly transmit through heterotrimeric G proteins, in the course of treatment with PEG-IFNα-RBV, release of multiple chemokines may involve heptahelical receptors coupled to G proteins [22]. Therefore, polymorphisms in related G proteins could also influence the signaling cascade in the treatment response.

In the present study, we investigated the association between single nucleotide polymorphisms (SNPs) in the *GNB1* gene and their susceptibility to RVR in Chinese patients in Taiwan receiving PEG-IFNα-RBV treatment. Our results support *GNB1* as a potential candidate gene for predictive therapeutic outcomes of HCV-1 and HCV-2 infected patients.

## Methods

### Patients

In the present study, 265 HCV-1 infected patients and 195 HCV-2 infected patients at China Medical University Hospital, Taichung, Taiwan were enrolled and actively observed. Diagnosis of HCV infection was based on persistent elevation of serum transaminase levels for at least 6 months, serum anti-HCV-positivity, and consistent detection of serum HCV RNA. Patients with hepatitis B surface antigen and antibodies or human immunodeficiency virus 1 and 2 positivity were excluded from this study. The enrolled patients were subcutaneously injected with PEG-IFNα (PEG-IFNα-2b, Peg-Intron, Schering-Plough, Kenilworth, NJ, USA) at a dose of 1.5 μg/kg/week in combination with weight-adapted doses of oral RBV (<60 kg, 800 mg/day; 60–75 kg, 1000 mg/day; and >75 kg, 1200 mg/day) for 48 weeks (HCV-1) or 24 weeks (HCV-2). All subjects provided informed consent. The study protocol was approved by the chairman of the Ethics Committee of China Medical University Hospital, Taichung, Taiwan, and was conducted in accordance with the Declaration of Helsinki.

### SNP selection

Selection of representative *GNB1* SNPs was based on SNP genotype information downloaded in December 2008 from the HapMap Chinese Han in Beijing (CHB) + JPT population. HapMap genotypes were further analyzed via Haploview software (version 4.2). Tag SNPs were selected using the Tagger function according to the following criteria: (1) minor allele frequency (MAF) in the HapMap CHB + JPT population >0.10, and (2) a ≥0.6 genotyping score (recommended by the manufacturer, Illumina, Inc., San Diego, CA) to reach a successful genotyping rate. Four SNPs in the *GNB1* gene met these criteria and were selected: rs10907185 (S1) (A/G in intron 7), rs6603797 (S2) (C/T in intron 2), rs4648727 (S3) (A/C in intron 1), and rs12126768 (S4) (G/T in intron 1).

### HCV genotyping and RNA measurements

HCV genotyping according to the classification of Simmonds *et al.*[23] was performed by reverse hybridization assay (INNO LiPA HCV-II; Innogenetics, Gent, Belgium). Virologic response was assessed using a qualitative HCV RNA assays with a low sensitivity of 30–50 IU/mL (HCV Amplicor™ 2.0, Roche Diagnostics, Branchburg, NJ). All HCV RNA level results are reported in copies/mL. According to the qualitative HCV RNA results, patients were defined as (1) rapid virological responders (RVRs, HCV RNA negative at week 4 of treatment), denoted as RVR (+), or (2) non-rapid virological responders (non-RVRs, HCV RNA positive at week 4 of treatment), denoted as RVR (−). The term “flat responder” refers to the population that showed no variation in the HCV RNA after receiving the SOC treatment. Flat responders were not present among the patients enrolled in this study because the change in the HCV RNA was unique for each patient; any patient who did not fit the RVR criteria was classified into the RVR (−) group. Therefore, all subjects were classified as RVR (+) or RVR (−).

### Genomic DNA extraction and genotyping

Genomic DNA was isolated from the peripheral blood of all participants by using a commercial kit (Genomic DNA kit; QIAGEN, Valencia, CA) according to the manufacturer’s instructions. All 4 SNPs in *GNB1* were genotyped using an allele-specific extension method and ligation assay according to the manufacturer’s instructions (Illumina, San Diego, CA).

### Statistical analysis

The association between each categories was assessed by the χ^2^ test. Genotype and allele frequencies in RVR (+) and RVR (−) subjects were compared, and odds ratios (ORs) with 95% confidence intervals (CIs) were estimated by unconditional logistic regression. Variables such as age, body mass index (BMI), aspartate aminotransferase (AST), alanine aminotransferase (ALT), platelet (PLT), and viral load were estimated by the Mann–Whitney *U* test. The differences among genotypes and viral loads were estimated by the Kruskal–Wallis test for multiple groups. All statistical analyses were performed using SPSS version 20.0 for Windows (Chicago, IL). Results with a *P* value less than 0.05 were considered statistically significant. Screening and construction of linkage disequilibrium (LD) plots were performed using Haploview (version 4.2). Haplotype analysis with sliding windows and Hardy–Weinberg equilibrium (HWE) was inferred using PLINK (version 1.07; http://pngu.mgh.harvard.edu/purcell/plink/) [24].

## Results

### Patient characteristics

The characteristics and clinical information for the HCV infected participants by HCV genotype are shown in Table 1. A total of 265 HCV-1 infected patients and 195 HCV-2 infected patients were enrolled. The overall RVR rates of HCV-1 and HCV-2 infected patients were 40.8% and 81.0%, respectively. The distribution of the *GNB1* phenotype among the sexes, age at study entry, BMI, degree of inflammatory activity, and stage of fibrosis did not differ significantly according to RVR (+/−) in the HCV-1 and HCV-2 groups. However, viral load at the start of treatment differed significantly between the RVR (+) and RVR (−) patients in the HCV-1 group (*P* < 0.0001). The viral loads of HCV-1 infected patients with rs10907185, rs6603797, rs4648727, and rs12126768 genotypes did not differ significantly (*P* = 0.270, *P* = 0.559, *P* = 0.952, and *P* = 0.414, respectively).

**Table 1:** Characteristics of HCV genotype 1 and 2 infected patients receiving PEG-IFNα-RBV therapy

	**HCV genotype 1 (HCV-1)**				**HCV genotype 2 (HCV-2)**			
	**Total**	**RVR (+)**	**RVR (−)**	***P*value**	**Total**	**RVR (+)**	**RVR (−)**	***P*value**
Number of patients	265	108	157	-	195	158	37	-
Sex (males/females)	129/136	55/53	74/83	0.545	88/107	75/83	13/24	0.176
Age (mean ± SD)	52.17 ± 10.27	50.96 ± 10.97	52.99 ± 9.71	0.172^a^	51.62 ± 10.89	51.08 ± 10.92	53.89 ± 10.62	0.095^a^
BMI (mean ± SD)	24.6 ± 3.1	24.6 ± 3.0	24.6 ± 3.2	0.971^a^	24.5 ± 3.5	24.5 ± 3.7	24.5 ± 3.1	0.933^a^
Degree of inflammatory activity (A0/A1-3)	35/230	10/98	25/132	0.115^b^	22/173	19/139	3/34	0.498 ^b^
Stage of fibrosis (F0/F1-4)	16/249	3/105	13/144	0.065 ^b^	10/185	9/149	1/36	0.457 ^b^
AST (U/L) (mean ± SD)		83.5 ± 57.1	80.7 ± 45.1	0.988 ^a^		85.7 ± 65.5	75.2 ± 39.1	0.677 ^a^
ALT (U/L) (mean ± SD)		132.2 ± 93.5	102.4 ± 57.6	0.018 ^a^		122.9 ± 108	101.9 ± 60.7	0.329 ^a^
PLT (×10^3^/μl) (mean ± SD)		174.6 ± 62.3	163.7 ± 55.7	0.146 ^a^		173.6 ± 53.5	155.5 ± 50.1	0.041 ^a^
Viral load (× 10^6^)	12.1 ± 16.4	7.0 ± 10.3	16.0 ± 18.8	<0.0001^a^	11.0 ± 19.0	10.0 ± 18.2	15.3 ± 21.8	0.217^a^

Abbreviations: RVR, rapid virological response; BMI, body mass index; AST, aspartate aminotransferase; ALT, alanine aminotransferase; PLT, platelet.

^a^ Calculated by the Mann–Whitney *U* test.

^b^ Calculated by the by *χ*^2^ test.

All 4 *GNB1* SNPs genotyped in this study were in Hardy–Weinberg equilibrium (HWE) (*P* > 0.05). This indicated that there was no population stratification bias or genotyping error in our anticipated subjects. Information on the SNPs, including chromosome position, HWE, and minor allele frequencies (MAF) is listed in Table 2.

**Table 2:** **Four single nucleotide polymorphisms in the *GNB1 *gene in 265 HCV-1 and 195 HCV-2 infected patients receiving PEG-IFNα-RBV therapy with or without a RVR in a Chinese population in Taiwan**

**SNP**	**Position in*GNB1***	**Chromosome position**^**a**^	**Alleles**	**HCV-1**			**HCV-2**		
				**HWE (*P*value)**	**MAF**		**HWE (*P*value)**	**MAF**	
					**RVR (+)**	**RVR (−)**		**RVR (+)**	**RVR (−)**
rs10907185 (S1)	Intron 7	1733219	A/G	0.2132	0.3009	0.2548	0.465	0.2785	0.2162
rs6603797 (S2)	Intron 2	1765583	C/T	0.5854	0.1065	0.1401	0.4007	0.0981	0.0946
rs4648727 (S3)	Intron 1	1776269	A/C	0.1309	0.3241	0.3429	0.8709	0.3513	0.2361
rs12126768 (S4)	Intron 1	1778090	G/T	0.7439	0.2083	0.2756	1	0.2373	0.2162

Abbreviations: SNP, single nucleotide polymorphism; HWE, Hardy–Weinberg equilibrium; MAF, minor allele frequency.

^a^ Chromosome positions refer to the sequence in the NCBI database (build 37.3).

### Association between tagging SNPs of *GNB1* and therapeutic response, RVR

The genotype frequencies of each SNP showing responsiveness to PEG-IFNα-RBV therapy are shown in Table 3. In the genotype association tests, none of the genotypes was associated with RVR in HCV-1 infected patients (Table 3). However, the combination of genotypes G/G and G/T of rs12126768 was significantly inversely correlated with RVR responsiveness (*P* = 0.0330, OR = 0.58, 95% CI = 0.35, 0.96). In HCV-2 infected patients, the polymorphism at position rs4648727 in the *GNB1* gene was statistically associated with RVR (*P* = 0.0194). For the A/A + A/C versus C/C genotype, we calculated an increased crude OR of 2.67 (95% CI = 1.26, 5.65; *P* = 0.0089) for RVR (+) versus RVR (−). The association of rs12126768 genotypes with RVR remained significant in the HCV-2 infected group (*P* = 0.0436). Therefore, HCV infected individuals with the *GNB1* rs4648727 C/C and rs12126768 G/G genotypes may be at increased risk being non-responsive to PEG-IFNα-RBV treatment.

**Table 3:** **Genotype frequencies of *GNB1 *single nucleotide polymorphisms (SNPs) in HCV-1 and HCV-2 infected patients receiving PEG-IFNα-RBV therapy with and without a RVR in a Chinese population in Taiwan**

**HCV-1**					**HCV-2**				
**SNP ID**	**RVR (+) (N = 108) N (%)**	**RVR (−) (N = 157) N (%)**	***P*value**	**OR (95% CI)**	**SNP ID**	**RVR (+) (N = 158) N (%)**	**RVR (−) (N = 37) N (%)**	***P*value**	**OR (95% CI)**
**rs10907185**					**rs10907185**				
A/A	10 (9.2)	5 (3.2)		3.09 (1.00, 9.56)	A/A	13 (8.3)	3 (8.1)		1.25 (0.33, 4.76)
A/G	45 (41.7)	70 (44.6)		0.99 (0.60, 1.66)	A/G	62 (39.2)	10 (27.0)		1.79 (0.80, 4.02)
G/G	53 (49.1)	82 (52.2)	0.1096	1	G/G	83 (52.5)	24 (64.9)	0.3601	1
A/A + A/G	55 (50.9)	75 (47.8)	0.6137	1.13 (0.69, 1.85)	A/A + A/G	75 (47.5)	13 (35.1)	0.1748	1.67 (0.79, 3.51)
**rs6603797**					**rs6603797**				
C/C	87 (80.5)	116 (73.9)		1.13 (0.18, 6.88)	C/C	129 (81.6)	31 (83.8)		2.08 (0.18, 23.69)
C/T	19 (17.6)	38 (24.2)		0.75 (0.12, 4.88)	C/T	27 (17.1)	5 (13.5)		2.70 (0.20, 35.75)
T/T	2 (1.9)	3 (1.9)	0.4332	1	T/T	2 (1.3)	1 (2.7)	0.7216	1
C/C + C/T	106 (98.1)	154 (98.1)	0.9723	1.03 (0.17, 6.29)	C/C + C/T	156 (98.7)	36 (97.3)	0.5227	2.17 (0.19, 24.55)
**rs4648727**^**a**^					**rs4648727**^**a**^				
A/A	10 (9.3)	14 (9.0)		0.94 (0.38, 2.29)	A/A	16 (10.1)	4 (11.1)		1.46 (0.44, 4.83)
A/C	50 (46.3)	79 (50.6)		0.83 (0.50, 1.39)	A/C	79 (50.0)	9 (25.0)		3.20 (1.39, 7.41)
C/C	48 (44.4)	63 (40.4)	0.7779	1	C/C	63 (39.9)	23 (63.9)	0.0194*	1
A/A + A/C	60 (55.6)	93 (59.6)	0.5112	0.86 (0.52, 1.41)	A/A + A/C	95 (60.1)	13 (36.1)	0.0089*	2.67 (1.26, 5.65)
**rs12126768**^**a**^					**rs12126768**				
G/G	6 (5.5)	9 (5.8)		0.76 (0.26, 2.25)	G/G	6 (3.8)	4 (10.8)		0.42 (0.11, 1.61)
G/T	33 (30.6)	68 (43.6)		0.56 (0.33, 0.94)	G/T	63 (39.9)	8 (21.6)		2.21 (0.94, 5.22)
T/T	69 (63.9)	79 (50.6)	0.0891	1	T/T	89 (56.3)	25 (67.6)	0.0436*	1
G/G + G/T	39 (36.1)	77 (49.4)	0.0330*	0.58 (0.35, 0.96)	G/G + G/T	69 (43.7)	12 (32.4)	0.2118	1.62 (0.76, 3.44)

^a^: Contains 1 missing data point in the RVR (−) group.

Abbreviations: SNP, single nucleotide polymorphism; RVR, rapid virological response; OR, odds ratio; CI, confidence interval.

Genotype frequencies were determined by *χ*^2^ test using 2 × 3 or 2 × 2 tables as appropriate. Odds ratios and 95% CI per genotype were estimated by unconditional logistic regression. *P* values less than 0.05 were considered statistically significant, and are denoted with an asterisk.

Although the distribution of some *GNB1* genotypes differed significantly between RVR (+) and RVR (−), the distribution of the allele frequencies of the SNPs did not differ significantly between these 2 groups (Table 4). We subsequently stratified patients by gender and HCV genotype (HCV-1 or HCV-2), and investigated *GNB1* genotype-dependent treatment responses. Table 5 summarizes the genotype distributions of the *GNB1* polymorphisms stratified by gender. The genotypes of SNP rs4648727 and rs12126768 were significantly associated with RVR in HCV-2 infected females (*P* = 0.0202 and *P* = 0.0350, respectively). However, no gender effects were associated with genotype responsiveness in the HCV-1 infected population. The allele frequencies of all the SNPs did not differ significantly between the males and females in either group (Table 6).

**Table 4:** **Allele frequencies of *GNB1 *single nucleotide polymorphisms in HCV-1 and HCV-2 infected patients receiving PEG-IFNα-RBV therapy with and without a RVR in a Chinese population in Taiwan**

**HCV-1**					**HCV-2**				
**SNP ID**	**RVR (+) (N = 108) N (%)**	**RVR (−) (N = 157) N (%)**	***P*value**	**OR (95% CI)**	**SNP ID**	**RVR (+) (N = 158) N (%)**	**RVR (−) (N = 37) N (%)**	***P*value**	**OR (95% CI)**
**rs10907185**					**rs10907185**				
A allele	65 (30.0)	80 (25.5)		1.26 (0.86, 1.85)	A allele	88 (27.8)	16 (21.6)		1.40 (0.76, 2.56)
G allele	151 (70.0)	234 (74.5)	0.2416	1	G allele	228 (72.2)	58 (78.4)	0.2756	1
**rs6603797**					**rs6603797**				
C allele	193 (89.4)	270 (86.0)		1.37 (0.80, 2.34)	C allele	285 (90.2)	67 (90.5)		0.96 (0.41, 2.28)
T allele	23 (10.6)	44 (14.0)	0.2521	1	T allele	31 (9.8)	7 (9.5)	0.9270	1
**rs4648727**^**a**^					**rs4648727**^**a**^				
A allele	70 (32.4)	107 (34.3)		0.92 (0.64, 1.33)	A allele	111 (35.1)	17 (23.6)		1.75 (0.97, 3.16)
C allele	146 (67.6)	205 (65.7)	0.6515	1	C allele	205 (64.9)	55 (76.4)	0.0607	1
**rs12126768**^**a**^					**rs12126768**				
G allele	45 (20.8)	86 (27.6)		0.69 (0.46, 1.04)	G allele	75 (23.7)	16 (21.6)		1.13 (0.61, 2.08)
T allele	171 (79.2)	226 (72.4)	0.0783	1	T allele	241 (76.3)	58 (78.4)	0.6989	1

^a^: Contains 1 missing data point in the RVR (−) group.

Abbreviations: SNP, single nucleotide polymorphism; RVR, rapid virological response; OR, odds ratio; CI, confidence interval.

Allele frequencies were determined by *χ*^2^ test using 2 × 2 tables. Odds ratios and 95% CI per allele were estimated by unconditional logistic regression. *P* values less than 0.05 were considered statistically significant.

**Table 5:** **Genotype frequencies of *GNB1 *single nucleotide polymorphisms stratified by gender in HCV-1 and HCV-2 infected patients receiving PEG-IFNα-RBV therapy with and without a RVR in a Chinese population in Taiwan**

**HCV-1**					**HCV-2**				
**SNP ID**	**RVR (+) N (%)**	**RVR (−) N (%)**	***P*value**	**OR (95% CI)**	**SNP ID**	**RVR (+) N (%)**	**RVR (−) N (%)**	***P*value**	**OR (95% CI)**
**rs10907185**					**rs10907185**				
**Males**					**Males**				
A/A	6 (10.9)	2 (2.8)		4.32 (0.81, 23.17)	A/A	8 (10.7)	0 (0.0)		-
A/G	24 (43.6)	36 (48.6)		0.96 (0.46, 1.98)	A/G	27 (36.0)	6 (46.2)		0.79 (0.24, 2.60)
G/G	25 (45.5)	36 (48.6)	0.1600	1	G/G	40 (53.3)	7 (53.8)	0.4292	1
A/A + A/G	30 (54.5)	38 (51.4)	0.7193	1.14 (0.56, 2.29)	A/A + A/G	35 (46.7)	6 (46.2)	0.9727	1.02 (0.31, 3.33)
**Females**					**Females**				
A/A	4 (7.6)	3 (3.6)		2.19 (0.46, 10.52)	A/A	5 (6.0)	3 (12.5)		0.66 (0.14, 3.07)
A/G	21 (39.6)	34 (41.0)		1.01 (0.49, 2.08)	A/G	35 (42.2)	4 (16.7)		3.46 (1.07, 11.22)
G/G	28 (52.8)	46 (55.4)	0.5986	1	G/G	43 (51.8)	17 (70.8)	0.0618	1
A/A + A/G	25 (47.2)	37 (44.6)	0.7673	1.11 (0.56, 2.22)	A/A + A/G	40 (48.2)	7 (29.2)	0.0981	2.26 (0.85, 6.02)
**rs6603797**					**rs6603797**				
**Males**					**Males**				
C/C	45 (81.8)	50 (67.6)		-	C/C	60 (80.0)	12 (92.3)		-
C/T	9 (16.4)	24 (32.4)		-	C/T	15 (20.0)	1 (7.7)		-
T/T	1 (1.8)	0 (0.0)	0.0672	1	T/T	0 (0.0)	0 (0.0)	-	1
C/C + C/T	54 (98.2)	74 (100.0)	0.2442	-	C/C + C/T	75 (100.0)	13 (100.0)	-	-
**Females**					**Females**				
C/C	42 (79.2)	66 (79.5)		1.91 (0.19, 18.97)	C/C	69 (83.1)	19 (79.2)		1.82 (0.16, 21.12)
C/T	10 (18.9)	14 (16.9)		2.14 (0.19, 23.72)	C/T	12 (14.5)	4 (16.6)		1.50 (0.11, 21.31)
T/T	1 (1.9)	3 (3.6)	0.8179	1	T/T	2 (2.4)	1 (4.2)	0.8601	1
C/C + C/T	52 (98.1)	80 (96.4)	0.5609	1.95 (0.20, 19.26)	C/C + C/T	81 (97.6)	23 (95.8)	0.6461	1.76 (0.15, 20.30)
**rs4648727**					**rs4648727**				
**Males**					**Males**^**a**^				
A/A	4 (7.3)	5 (6.8)		1.20 (0.29, 5.02)	A/A	9 (12.0)	0 (0.0)		-
A/C	31 (56.4)	39 (52.7)		1.19 (0.57, 2.49)	A/C	39 (52.0)	5 (41.7)		2.02 (0.58, 7.05)
C/C	20 (36.3)	30 (40.5)	0.8905	1	C/C	27 (36.0)	7 (58.3)	0.2255	1
A/A + A/C	35 (63.6)	44 (59.5)	0.6301	1.19 (0.58, 2.45)	A/A + A/C	48 (64.0)	5 (41.7)	0.1410	2.49 (0.72, 8.61)
**Females**^**a**^					**Females**				
A/A	6 (11.4)	9 (11.0)		0.79 (0.25, 2.48)	A/A	7 (8.4)	4 (16.7)		0.78 (0.20, 3.04)
A/C	19 (35.8)	40 (48.8)		0.56 (0.27, 1.18)	A/C	40 (48.2)	4 (16.7)		4.44 (1.36, 14.53)
C/C	28 (52.8)	33 (40.2)	0.3067	1	C/C	36 (43.4)	16 (66.6)	0.0202*	1
A/A + A/C	25 (47.2)	49 (59.8)	0.1513	0.60 (0.30, 1.21)	A/A + A/C	47 (56.6)	8 (33.3)	0.0443*	2.61 (1.01, 6.77)
**rs12126768**					**rs12126768**				
**Males**					**Males**				
G/G	3 (5.5)	3 (4.1)		1.06 (0.20, 5.59)	G/G	2 (2.7)	0 (0.0)		-
G/T	17 (30.9)	34 (45.9)		0.53 (0.25, 1.11)	G/T	31 (41.3)	4 (30.8)		1.66 (0.47, 5.89)
T/T	35 (63.6)	37 (50.0)	0.2244	1	T/T	42 (56.0)	9 (69.2)	0.6089	1
G/G + G/T	20 (36.4)	37 (50.0)	0.1230	0.57 (0.28, 1.17)	G/G + G/T	33 (44.0)	4 (30.8)	0.3723	1.77 (0.50, 6.25)
**Females**^**a**^					**Females**				
G/G	3 (5.7)	6 (7.3)		0.62 (0.14, 2.65)	G/G	4 (4.8)	4 (16.7)		0.34 (0.08, 1.52)
G/T	16 (30.2)	34 (41.5)		0.58 (0.28, 1.23)	G/T	32 (38.6)	4 (16.7)		2.72 (0.83, 8.90)
T/T	34 (64.1)	42 (51.2)	0.3339	1	T/T	47 (56.6)	16 (66.6)	0.0350*	1
G/G + G/T	19 (35.8)	40 (48.8)	0.1391	0.59 (0.29, 1.19)	G/G + G/T	36 (43.4)	8 (33.4)	0.3786	1.53 (0.59, 3.97)

^a^: Contains 1 missing data point in the RVR (−) group.

Abbreviations: SNP, single nucleotide polymorphism; RVR, rapid virological response; OR, odds ratio; CI, confidence interval.

Genotype frequencies were determined by *χ*^2^ test using 2 × 3 or 2 × 2 tables as appropriate. Odds ratios and 95% CI per genotype were estimated by unconditional logistic regression. *P* values less than 0.05 were considered statistically significant, and are denoted with an asterisk.

**Table 6:** **Allele frequencies of *GNB1 *single nucleotide polymorphisms stratified by gender in HCV-1- and HCV-2-infected patients receiving PEG-IFNα-RBV therapy with and without RVR in a Chinese population in Taiwan**

**HCV-1**					**HCV-2**				
**SNP ID**	**RVR (+) N (%)**	**RVR (−) N (%)**	***P*value**	**OR (95% CI)**	**SNP ID**	**RVR (+) N (%)**	**RVR (−) N (%)**	***P*value**	**OR (95% CI)**
**Males**									
**rs10907185**					**rs10907185**				
A allele	36 (32.7)	40 (27.0)		1.31 (0.77, 2.25)	A allele	43 (28.7)	6 (23.1)		1.34 (0.50, 3.56)
G allele	74 (67.3)	108 (73.0)	0.3206	1	G allele	107 (71.3)	20 (76.9)	0.5572	1
**rs6603797**					**rs6603797**				
C allele	99 (90.0)	124 (83.8)		1.74 (0.81, 3.73)	C allele	135 (90.0)	25 (96.2)		0.36 (0.05, 2.85)
T allele	11 (10.0)	24 (16.2)	0.1493	1	T allele	15 (10.0)	1 (3.8)	0.3136	1
**rs4648727**					**rs4648727**^**a**^				
A allele	39 (35.5)	49 (33.1)		1.11 (0.66, 1.87)	A allele	57 (38.0)	5 (20.8)		2.33 (0.82, 6.58)
C allele	71 (64.5)	99 (66.9)	0.6942	1	C allele	93 (62.0)	19 (79.2)	0.1030	1
**rs12126768**					**rs12126768**				
G allele	23 (20.9)	40 (27.0)		0.71 (0.40, 1.28)	G allele	35 (23.3)	4 (15.4)		1.67 (0.54, 5.18)
T allele	87 (79.1)	108 (73.0)	0.2580	1	T allele	115 (76.7)	22 (84.6)	0.3676	1
**Females**									
**rs10907185**					**rs10907185**				
A allele	29 (27.4)	40 (24.1)		1.19 (0.68, 2.07)	A allele	45 (27.1)	10 (20.8)		1.41 (0.65, 3.07)
G allele	77 (72.6)	126 (75.9)	0.5465	1	G allele	121 (72.9)	38 (79.2)	0.3809	1
**rs6603797**					**rs6603797**				
C allele	94 (88.7)	146 (88.0)		1.07 (0.50, 2.30)	C allele	150 (90.4)	42 (87.5)		1.34 (0.49, 3.64)
T allele	12 (11.3)	20 (12.0)	0.8559	1	T allele	16 (9.6)	6 (12.5)	0.5653	1
**rs4648727**^**a**^					**rs4648727**				
A allele	31 (29.2)	58 (35.4)		0.76 (0.45, 1.28)	A allele	54 (32.5)	12 (25.0)		1.45 (0.70, 3.00)
C allele	75 (70.8)	106 (64.6)	0.2961	1	C allele	112 (67.5)	36 (75.0)	0.3198	1
**rs12126768**^**a**^					**rs12126768**				
G allele	22 (20.8)	46 (28.0)		0.67 (0.38, 1.20)	G allele	40 (24.1)	12 (25.0)		0.95 (0.45, 2.00)
T allele	84 (79.2)	118 (72.0)	0.1776	1	T allele	126 (75.9)	36 (75.0)	0.8977	1

^a^: Contains 1 missing data point in the RVR (−) group.

Abbreviations: SNP, single nucleotide polymorphism; RVR, rapid virological response; OR, odds ratio; CI, confidence interval.

Allele frequencies were determined by the *χ*^2^ test using 2 × 2 tables. Odds ratios and 95% CI per genotype were estimated by unconditional logistic regression. *P* values less than 0.05 were considered statistically significant.

**Figure 1:**
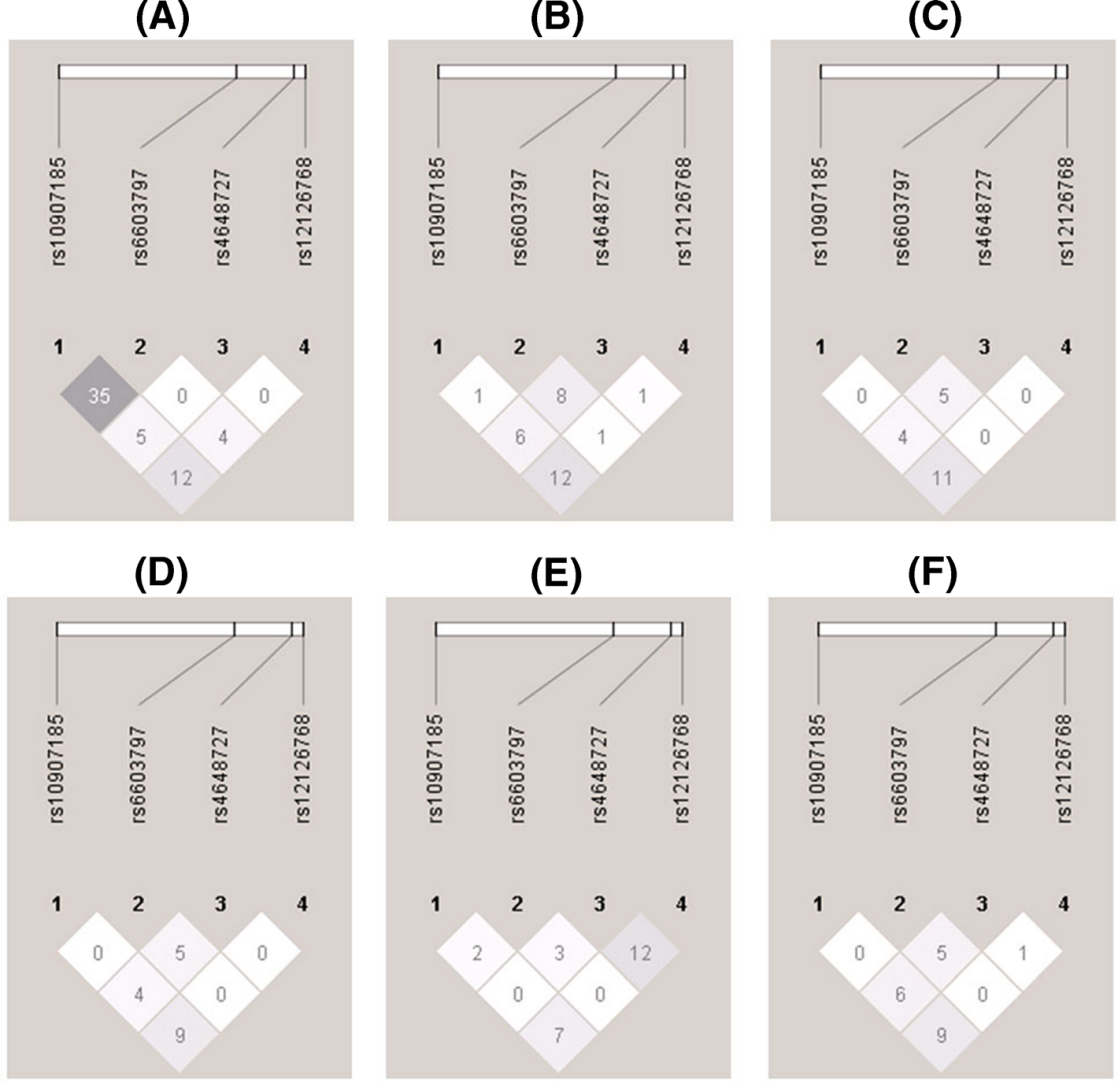
**Linkage disequilibrium plot of the analyzed SNPs in the *GNB1 *gene.** The figure shows the output of a Haploview (version 4.2) linkage disequilibrium plot where each square (r^2^ values written within the box correspond to r^2^ values × 100 as a linkage disequilibrium measure range) represents a pairwise linkage disequilibrium relationship between the 2 SNPs. Darkest colored squares indicate high linkage disequilibrium (r^2^ = 1); medium colored squares indicate r^2^ values between 0 and 1; and the lightest colored squares indicate low linkage disequilibrium (r^2^ = 0). The figure depicts the linkage disequilibrium pattern in (**A**) all HCV genotype 1 (HCV-1) infected patients; (**B**) HCV-1 infected patients receiving SOC therapy with RVR (−); (**C**) HCV-1-infected patients receiving SOC therapy with RVR (+); (**D**) all HCV genotype 2 (HCV-2) infected patients; (**E**) HCV-2 infected patients receiving SOC therapy with RVR (−); (**F**) HCV-2 infected patients receiving SOC therapy with RVR (+).

For LD analysis, our results indicated the existence of a low degree of pairwise LD among these SNPs in HCV-1 and HCV-2 infected populations with or without RVR. A graphical summary of the LDs (r^2^ values) among the tested SNPs at different loci is shown in Figure 1. Four tag SNPs were selected and designated in a sequential order.

### Frequencies of *GNB1* haplotypes and their association with RVR

We compared the haplotype frequencies and treatment responses adjusting for gender as a covariate to avoid potential confounding effects using the PLINK program as shown in Table 7. Global analyses of haplotypes were performed by sliding window mode to examine all the possible sizes of the haplotypes (number of SNPs per haplotype). We found that there were 10 sliding windows, 2 of which were significantly associated with RVR (omnibus test *P* < 0.05) in HCV-1 infected patients.

**Table 7:** **Summary of exhaustive haplotype analyses based on sex-adjusted omnibus tests for sliding windows of all possible sizes across 4 *GNB1 *SNPs**

**Sliding window (SW)**		**Most significant result**			
		**HCV Genotype**			
		**1**	**2**	**1**	**2**
**SNPs/SW**	**No. of SW**	**SW**	**SW**	***P*value**	***P*value**
1	4	-	-	-	-
2	3	S1.S2	S3.S4	0.0208*	0.0546
3	2	S2.S4	S2.S4	0.0540	0.1099
4	1	S1.S4	S1.S4	0.0397*	0.1585

S1: rs10907185.

S2: rs6603797.

S3: rs4648727.

S4: rs12126768.

The overall global test, details, and haplotype frequencies are listed in Table 8. In HCV-1 infected patients, haplotype AC, the window S1-S2, gave the most impressive *P* value for the omnibus test. However, it did not play a significant role in HCV-2 infected patients. Haplotype-specific analyses showed that the CAT haplotypes (S2-S3-S4) might increase the rate of RVR (*P* = 0.0265; OR = 4.50) when compared to the RVR (−) groups, especially in the HCV-2 infected population. The window S1-S2-S3-S4 with the ACAT haplotypes was significantly positively associated with a higher rate of RVR in both HCV-1 and HCV-2 infected patients (OR = 2.01, *P* = 0.0261 and OR = 4.54, *P* = 0.0253, respectively). Furthermore, the results showed that HCV-1 and HCV-2 infected patients with therapeutic responses had the ACAT haplotypes, and thus the ACAT haplotype appeared more frequently in RVR (+) patients than in RVR (−) patients. Therefore, in HCV-1 or HCV-2 infected individuals, haplotype-specific analysis showed that the haplotype ACAT (S1-S2-S3-S4) was associated with an increase in the RVR rate. This observation suggests that the haplotype ACAT may play a role in the response to PEG-IFNα-RBV treatment.

**Table 8:** Details of sex-adjusted haplotype frequency analysis for 2-SNP, 3-SNP, and 4-SNP windows showing the most significant results among all possible sliding windows

**HCV-1**				
**Haplotypes**	**RVR (+)**	**RVR (−)**	**OR**	***P*value**
rs10907185-rs6603797 (S1-S2)				
OMNIBUS	-	-	-	0.0208*
AC	0.2	0.1158	1.91	0.0078*
rs6603797-rs4648727-rs12126768 (S2-S3-S4)				
OMNIBUS	-	-	-	0.054
CAT	0.1157	0.0673	1.81	0.0524
rs10907185-rs6603797-rs4648727-rs12126768 (S1-S2-S3-S4)				
OMNIBUS	-	-	-	0.0397*
ACAT	0.1163	0.0615	2.01	0.0261*
**HCV-2**				
**Haplotypes**	**RVR (+)**	**RVR (−)**	**OR**	***P*value**
rs4648727-rs12126768 (S3-S4)				
OMNIBUS	-	-	-	0.0546
AT	0.1139	0.0278	4.50	0.0265*
rs6603797-rs4648727-rs12126768 (S2-S3-S4)				
OMNIBUS	-	-	-	0.1099
CAT	0.1139	0.0278	4.50	0.0265*
rs10907185-rs6603797-rs4648727-rs12126768 (S1-S2-S3-S4)				
OMNIBUS	-	-	-	0.1585
ACAT	0.1149	0.0278	4.54	0.0253*

## Discussion

We examined the association between related genes and the efficacy of SOC therapy and identified the *GNB1* gene on chromosome 1 as a new candidate susceptibility gene. In this study, we found that the SNPs rs4648727 and rs12126768 in the introns of *GNB1* may be associated with the rate of RVR to PEG-IFNα-RBV treatment. In addition, we found that 1 *GNB1* haplotype (ACAT), which is a combination of the set of SNPs in this gene, was statistically associated with RVR. Clinical association studies showed that the *GNB1* haplotype (ACAT) carriers were significantly associated with a higher archived rate of RVR (OR in the range of 1.81–4.54) in both HCV-1 and HCV-2 infected patients. This finding led us to the hypothesis that the treatment response to PEG-IFNα-RBV could, in part, be dependent on *GNB1*. The genotypes of *GNB1* were equally distributed in males and females, the viral load, age at study entry, BMI, and others clinical data did not differ significantly among the different genotypes in the HCV-1 and HCV-2 infected populations. Therefore, it is unlikely that specific *GNB1* genotypes predispose individuals to infection with HCV-1 and HCV-2 or contribute to spontaneous virus elimination.

Several reports have provided strong evidence that patients infected with HCV-1 have about a 50% (in Caucasians) and 80% (in African Americans) probability of a poor response toward PEG-IFNα-RBV treatment. In our study, the overall RVR rates were less than 45% and 85% in the HCV-1 and HCV-2 populations, respectively. Therefore, reliable prediction of a non-viral response in the beginning of treatment would avoid side effects and reduce the cost of treatment. Although, viral clearance has been strongly associated with various clinical features, for example, gender, age <40 years, low HCV RNA level before treatment, absence of liver cirrhosis, and HCV genotype 2/3 [25]. Many researchers are still focused on the identification of host genetic factors that may be related to clinical outcomes and providing custom therapy for HCV infection.

Previous studies have shown that the 825T allele of *GNB3* and its associated haplotypes are predictors of enhanced signal transduction via G proteins. The *GNB3* 825 C/C genotype is associated with non-response in HCV-1 infected patients [17,26]. Although the heterotrimeric transformation of G proteins is not directly involved in the interferon-signaling pathway, PEG-IFNα-RBV treatment, which may initiate multiple chemokine responses, may involve heptahelical receptors coupled to G proteins [22]. Therefore, polymorphism in genes encoding components of the chemokine system may be related to treatment outcomes. In this study, the exact mechanism by which *GNB1* genotypes are associated with decreased or increased G protein activation remains to be determined.

Although several virological responses have been used to predict SVR, on comparing the baseline characteristics, we observed that RVR increased and remained a strong predictor of SVR [27]. In the HCV-1 infected population, the SVR rate were significantly higher in the RVR (+) patients (89.8%) than in the RVR (−) patients (10.2%). On the other hand, in the HCV-2 infected population, the SVR rate was significantly higher in the RVR (+) group (94.3%) than in the RVR (−) group (5.7%). However, we did not find a significant association between *GNB1* SNPs and SVR. To estimate the sample sizes for this association test, which would ensure a maximum power of 80% at *P* < 0.05, we calculated the sample size by using the G*Power 3.1 software. In future studies, we plan to enroll a total of >1000 patients. Therefore, additional factors need to be incorporated to ensure a high likelihood for discriminating the patients with RVR who will achieve SVR from those who will not.

In 2 previous GWAS studies, they concluded that SNPs in or near the *IL-28B* gene strongly determined the outcome of HCV therapy [19,28-30]. The most significant SNP in their study groups was rs8099917, which was associated with SVR in European and Japanese patients. Interestingly, rs8099917 was not associated with the response to PEG-IFNα-RBV therapy in HCV-2/3 infected patients. They proposed that, the contributions of host genetic factors to HCV-2/3 clearance are relatively low compared with the contributions of host genetic factors to HCV-1 clearance because HCV-2/3 is more likely to be eliminated by SOC therapy. Our results demonstrated that the *GNB1* haplotype (ACAT) was significantly associated with a higher rate of RVR both in HCV-1 and HCV-2 infected populations. The significance of the genetic effect of *GNB1* in other ethnicities remains to be elucidated.

Although we did not analyze the functional effects of these intronic SNPs on the G protein, they were associated with SOC therapy outcome. Since the *GNB1* gene was weakly associated with therapeutic outcomes, the linkage among these *GNB1* polymorphisms and RVR/SVR should be confirmed in future studies with larger enrolled populations. The results of this study may provide novel information toward determining the exact response to PEG-IFNα-RBV.

## Conclusion

In conclusion, this study provided evidence that the *GNB1* gene polymorphisms are related to RVR in HCV-1 and HCV-2 infected patients. Furthermore, we determined that the *GNB1* haplotype (ACAT) plays a role in the clinical response. Therefore, we believe that *GNB1* may play an important role in activating the antiviral response prior to treatment.

## Competing interests

The authors declare that they have no competing interest.

## Authors’ contributions

YPL and LW designed and carried out most of the study. YPL wrote the manuscript and performed data analysis. FJT, WLL, NT, and DZH participated in clinical data and information collection. CYP and LW conceived and supervised the project and reviewed the manuscript. All authors contributed to and approved the final manuscript by providing constructive suggestions.

## Pre-publication history

The pre-publication history for this paper can be accessed here:

http://www.biomedcentral.com/1471-230X/12/167/prepub
